# 

**Published:** 1886-02

**Authors:** 


### NINTH INTERNATIONAL MEDICAL CONGRESS.

### SECTION ON DENTAL AND ORAL SURGERY.

As there seems to be some misapprehension in the minds of some, and earnest inquiry by others, as to the status of the Section on Dental and Oral Surgery, in the International Medical Congress, to be held in Washington, D. C., in 1887, it seems right and proper that some statement be now made in regard to the organization and progress of the work.

It is very generally known that the Section has been established and organized; the following officers have been appointed, viz: a President, one Vice-President, and two Secretaries.

Fourteen gentlemen of recognized ability and high professional standing, from various parts of the country have accepted positions upon the council, and have pledged themselves to do all they can to make this Section a success. At the next meeting of the Executive Committee, ten or twelve more names will be added to the Council, as may seem best. Much of the preliminary work in arranging the matters of the Section, has been in the main accomplished.

A programme—embracing the subjects of greatest interest to the profession of the world—has been outlined, and is now under consideration; and as soon as completed, the secretaries will open correspondence with the eminent men of the profession in Europe and America, relative to the work to be done. Quite a number have already indicated a desire to prepare papers, or at least take some part in the work.

We not only expect, but are assured. that this Section will receive the hearty support and co-operation of dental specialists, both at home and abroad; and with such manifestations, great hope is entertained that the Section will be eminently successful. We ask for it the co-operation of all who have the best interests of our profession at heart.

A circular will ere long be issued by the Executive Committee, giving the status of the preparatory work for the Congress.

J. TAFT,
President of Section D. O. S.

---

### UNIVERSITY OF CALIFORNIA.

The fourth annual commencement exercises of the College of Dentistry, of the University of California, were held in connection with those of the Medical Department, at the Grand Opera House, San Francisco, on Tuesday, November 10th, 1885, at 2 o'clock.

### To Readers and Correspondents.

Communications intended for publication, and exchange journals and papers should be addressed to the Editor of the American Journal of Dental Science, 259 N. Eutaw St., Baltimore, Md. All letters relating to the business of the Journal, or containing cash remittances, to be directed to Messrs. Snowden & Cowman. Articles for publication should be received by the editor at least three weeks previous to the first month on which the number in which it is designed for them to appear, is issued.

☞ The American Journal of Dental Science will contain fifty pages of reading matter in each number, making a volume of about *Six Hundred* pages,

EXCLUSIVE OF ADVERTISEMENTS.

